# Factors Associated With the Utilization of Outpatient Virtual Clinics: Retrospective Observational Study Using Multilevel Analysis

**DOI:** 10.2196/40288

**Published:** 2022-08-12

**Authors:** Yun-Hsuan Tzeng, Wei-Hsian Yin, Kuan-Chia Lin, Jeng Wei, Hao-Ren Liou, Hung-Ju Sung, Hui-Chu Lang

**Affiliations:** 1 Health Management Center Cheng Hsin General Hospital Taipei Taiwan; 2 School of Medicine National Yang Ming Chiao Tung University Taipei Taiwan; 3 Heart Center Cheng Hsin General Hospital Taipei Taiwan; 4 Institute of Hospital and Health Care Administration National Yang Ming Chiao Tung University Taipei Taiwan; 5 Community Medicine Research Center National Yang Ming Chiao Tung University Taipei Taiwan

**Keywords:** telemedicine, remote consultation, e-consult, virtual clinic, outpatient, virtual care, virtual consult, physicians, health policy, health care delivery, COVID-19, multilevel analysis, outpatient clinic, telehealth, virtual health, health care system, adoption, attitude, perception

## Abstract

**Background:**

Although the COVID-19 pandemic has accelerated the adoption of telemedicine and virtual consultations worldwide, complex factors that may affect the use of virtual clinics are still unclear.

**Objective:**

This study aims to identify factors associated with the utilization of virtual clinics in the experience of virtual clinic service implementation in Taiwan.

**Methods:**

We retrospectively analyzed a total of 187,742 outpatient visits (176,815, 94.2%, in-person visits and 10,927, 5.8%, virtual visits) completed at a large general hospital in Taipei City from May 19 to July 31, 2021, after rapid implementation of virtual outpatient clinic visits due to the COVID-19 pandemic. Data of patients’ demographic characteristics, disease type, physicians’ features, and specialties/departments were collected, and physicians’ opinions regarding virtual clinics were surveyed and evaluated using a 5-point Likert scale. Multilevel analysis was conducted to determine the factors associated with the utilization of virtual clinics.

**Results:**

Patient-/visit-, physician-, and department-level factors accounted for 67.5%, 11.1%, and 21.4% of the total variance in the utilization of virtual clinics, respectively. Female sex (odds ratio [OR] 1.27, 95% CI 1.22-1.33, *P*<.001); residing at a greater distance away from the hospital (OR 2.36, 95% CI 2.15-2.58 if distance>50 km, *P*<.001; OR 3.95, 95% CI 3.11-5.02 if extensive travel required, *P*<.001); reimbursement by the National Health Insurance (NHI; OR 7.29, 95% CI 5.71-9.30, *P*<.001); seeking care for a major chronic disease (OR 1.33, 95% CI 1.24-1.42, *P*<.001); the physician’s positive attitude toward virtual clinics (OR 1.50, 95% CI 1.16-1.93, *P*=.002); and visits within certain departments, including the heart center, psychiatry, and internal medicine (OR 2.55, 95% CI 1.46-4.46, *P*=.004), were positively associated with the utilization of virtual clinics. The patient’s age, the physician’s age, and the physician’s sex were not associated with the utilization of virtual clinics in our study.

**Conclusions:**

Our results show that in addition to previously demonstrated patient-level factors that may influence telemedicine use, including the patient’s sex and distance from the hospital, factors at the visit level (insurance type, disease type), physician level (physician’s attitude toward virtual clinics), and department level also contribute to the utilization of virtual clinics. Although there was a more than 300-fold increase in the number of virtual visits during the pandemic compared with the prepandemic period, the majority (176,815/187,742, 94.2%) of the outpatient visits were still in-person visits during the study period. Therefore, it is of great importance to understand the factors impacting the utilization of virtual clinics to accelerate the implementation of telemedicine. The findings of our study may help direct policymaking for expanding the use of virtual clinics, especially in countries struggling with the development and promotion of telemedicine virtual clinic services.

## Introduction

During the COVID-19 pandemic, countries worldwide relaxed restrictions on the utilization of telemedicine to reduce the contagion, which has resulted in the expansion of telemedicine in various clinical applications [1-3]. Owing to the severity of the disease outbreak, many countries have implemented strict strategies to restrict movement, such as lockdowns, travel constraints, and quarantine, and have implemented extensive use of telemedicine in place of in-person clinic visits in order to reduce disease exposure and the risk of infection among patients and medical staff [4,5].

Although some initial research on the provision of health care via virtual clinics (virtual consultations, teleconsultations) in the United States and the United Kingdom during the COVID-19 pandemic reported generally high patient and provider satisfaction [6-8], the acceptance of virtual visits appeared to vary widely across different subspecialties and patient populations. A previous study reported that only 32% of patients with head and neck cancer chose to have a virtual visit, even during the COVID-19 pandemic [9]. In different countries, due to differing degrees of technology penetration and COVID-19 outbreak severity, the acceptance of virtual clinics seems to vary. For example, a study conducted in Australia showed that only 61.7% of patients were satisfied with virtual visits, and less than 50% of patients expressed the desire to continue to use it in the future [10]. In addition, research on whether patient demographics are associated with the willingness to use virtual clinics has revealed conflicting results. Although some studies have shown that patients who are female and younger than 65 years of age are more likely to use digital health services [11,12], others have found that female patients are less likely to use virtual clinics [13,14]. A large study analyzing 231,596 visits across 1652 primary and specialty care practices in the United States found that patient sex is not associated with differences in the use of video visits, whereas the type of practice and clinician specialty are the main drivers of variation in telemedicine usage [15].

The inconsistency among these preliminary findings suggests that further research is needed to better identify factors that potentially impact the utilization of virtual health care. In addition, factors beyond patient demographics may also play a role in the utilization of virtual clinics, including disease chronicity, physician characteristics, the physician’s attitude toward virtual clinics, and the type of subspecialty. Understanding the impact of these factors may enable policymakers and health care providers to increase patients’ receptiveness to virtual health care and to expand the utilization of virtual clinics in the postpandemic era. This study aims to identify factors associated with the utilization of virtual clinics in the experience of virtual clinic service implementation in Taiwan during the COVID-19 pandemic. The findings may help direct future policy for promoting and expanding the use of virtual clinics.

## Methods

### Background Information and Study Design

Prior to the COVID-19 pandemic, telemedicine regulations in Taiwan were restrictive. Before 2018, only residents of outlying islands or distant mountainous areas with insufficient medical resources were allowed to use telemedicine consultations in disease diagnosis and treatment. After 2018, a few specific patients, such as overseas patients or those admitted to a family physician integrated care plan, were added to the telemedicine project. Therefore, most physicians and patients in Taiwan had never used virtual consultations prior to the COVID-19 pandemic. The Cheng Hsin General Hospital is an 800-bed hospital located in Taipei City, Taiwan. In the pre–COVID-19 pandemic time, the average volume of our outpatient clinic was approximately 100,000 visits per month. Due to restrictive prepandemic telemedicine regulations, the number of telemedicine consultations was limited at approximately 10 visits per month. During the COVID-19 pandemic, the Taiwan government relaxed telemedicine regulations, and virtual clinic visits became reimbursed under the National Health Insurance (NHI) for all patients beginning May 16, 2021. Our institution rapidly responded to the change in policy and initiated virtual outpatient clinics conducted via an integrated user-friendly smartphone application platform beginning May 19, 2021. Both virtual and in-person clinic services were made available to all our outpatients, and patients could easily book an appointment for either type of visit through the smartphone application. This special background gave us a good opportunity to test the acceptance and demand for virtual outpatient clinics by the general public and physicians of various specialties. Thus, we conducted this retrospective, cross-sectional study to determine factors associated with virtual clinic utilization.

### Data Source

Data were collected from 2 sources: (1) data from the hospital information system (HIS) and the electronic medical record (EMR) system of the Cheng Hsin General Hospital and (2) results of a physician survey. The data extracted from our HIS and EMR systems contained patient age, sex, and address of residence; visit date; visit type (in-person visit or virtual visit); insurance type (whether reimbursed by the NHI); principal diagnosis codes; the in-charge physician; and the age, sex, and medical specialty/department of the physician. The design and procedures of the physician survey are described in a separate section later.

### Study Sample

We collected data of all outpatient visits of the Cheng Hsin General Hospital between May 19 and July 31, 2021. This study period was chosen because it was just after implementation of our virtual clinic platform and was the peak period of virtual visits during that year. To compare the usage of virtual clinics among various specialties, including pediatrics and geriatrics, we included patients of all ages in our study. Data from a total of 197,534 outpatient visits during the study period were collected. We excluded all visits from the Department of Emergency (n=3099, 1.6%), the Department of Health Examination (n=1623, 0.8%), and visits for COVID-19 vaccination (n=5070, 2.6%) because virtual clinic services were not available in those departments. After applying the exclusion criteria, the final data set included 187,742 visits, with 176,815 (94.2%) in-person visits and 10,927 (5.8%) virtual visits completed in the outpatient department of 30 subspecialties during the study period. For in-person visits, patients came to the hospital as usual because there were no lockdown restrictions in place in Taiwan during that period. For virtual visits, physicians conducted video calls with patients at the scheduled appointment time using the integrated platform. All virtual visits were booked by patients themselves and conducted by in-charge physicians using the same smartphone application platform. Audio-only visits occurred under the condition of insufficient internet bandwidth or poor Wi-Fi signals, which resulted in a video call without screen images and only audio signals being transmitted. Since the determination of video or audio visits mainly depended on the internet condition, further subgroup analysis between video and audio visits was not performed.

### Physician Survey Design

To understand physicians' opinions on the implementation of virtual clinics and to evaluate the performance of our newly introduced virtual clinic platform to identify areas for future improvement, all full-time physicians who provide outpatient clinic services at our hospital were invited to complete an online service survey. The survey was conducted between September 9 and October 6, 2021.

In the current absence of a widely validated physician survey of telemedicine that met our purpose, we designed a service-specific questionnaire modified from previously published questionnaires [6,16-18] and followed recommendations on the use of telemedicine research surveys [19]. The survey evaluated the following elements: general attitude; reliability; confidence in diagnostic and therapeutic assessment; technique-specific elements, such as audio and video quality; platform-specific elements, such as function and design; efficiency; and satisfaction (see Multimedia Appendix 1). Physicians were asked to provide answers using a 5-point Likert scale (1=strongly disagree to 5=strongly agree). Finally, we allowed for comments and suggestions. A single question regarding each physician’s general attitude toward virtual clinics was recorded, and the response was analyzed for the study.

### Ethical Considerations

As this was a formal service evaluation, ethical approval was not required for this study. Nonetheless, all invited physicians were fully informed verbally of the aims of this survey and understood that their responses would be analyzed for the purpose of publication. Participation was voluntary, and consent from physicians was implied by participation in the survey. This study was approved by the Institutional Review Board of the Cheng Hsin General Hospital (#(916)110-62). The need for informed written consent was waived by the board, and approval was granted for informed verbal consent prior to data collection.

### Study Measures

The outcome of interest in our study was visit type (in-person visit vs virtual visit). For patient-level variables, we included patient characteristics that have been previously demonstrated to influence telemedicine use, including age, sex, and distance between the patient’s residence and the hospital. Sociodemographic variables, such as the marital status, highest education level, and income of the patient, were not included, because updated data of this type were not available in our HIS database. In addition, no data were available in our HIS database regarding the patients’ race/ethnicity or spoken language. The distance between a patient’s place of residence and the hospital was estimated using the patient address’ zip code and then grouped as a categorical variable (<20 km, 20-50 km, >50 km, outlying islands, and traveled >5 hours to reach the hospital). We also collected visit-level variables, including insurance/reimbursement type and the coding of principal diagnosis based on the *International Classification of Diseases 10th Revision* (ICD-10) from our HIS and EMR databases.

To investigate the association between disease type and the usage of virtual clinics and to examine the association of major chronic diseases with virtual clinic service, we used the classifications of chronic diseases defined by the Ministry of Health and Welfare of Taiwan and the chronic condition indicator for ICD-10 developed by the Agency for Healthcare Research and Quality of America [20] to categorize the diseases. As defined by the Ministry of Health and Welfare of Taiwan, prescription refills are allowed for 101 chronic diseases in 16 categories (Multimedia Appendix 2). First, we removed certain disease groups that may involve complicated disease conditions, diverse prognoses, and various purposes for visits, including malignant neoplasm, brain tumor, polyneuropathy, nerve root and plexus disorders, trigeminal neuralgia, spinal cord injury, peptic ulcer, colitis, cholangitis, nephritis, arthritis, dermatomyositis, osteomyelitis, osteoporosis, autoimmune disease, ocular disease, skin diseases, ear diseases, blood diseases, prostate and urination diseases, infectious diseases, congenital malformations, hemorrhoids, follow-up after organ transplantation, and menopause syndrome. Next, we removed certain diseases that had few cases in our study cohort, including endometriosis, leprosy, blackfoot disease, and polychlorinated biphenyl intoxication. Finally, we defined 10 types of major chronic and stable diseases (Table 1) to compare with other diseases. The data of the dictionary of the specific ICD-10 codes used to categorize these diseases are provided in Multimedia Appendix 3. For physician-level variables, the physician’s age and sex were obtained from the HIS database and the physician’s general attitude toward virtual clinics was obtained from the physician survey, as described before. Department-level variables, such as the medical specialty/department of the visit, were collected from our HIS database as well.

**Table 1:** Major chronic diseases according to ICD-10^a^.

Disease group	Visits (N=187,742), n (%)
1. Diabetes mellitus	30,680 (16.3)
2. Coronary artery disease	19,517 (10.4)
3. Hypertension	14,034 (7.5)
4. Chronic cardiac and arterial disease	11,387 (6.1)
5. Psychiatric disease and sleep disorder	9887 (5.3)
6. Cerebrovascular disease and other chronic neurologic diseases	7477 (4.0)
7. Chronic respiratory disease	5196 (2.8)
8. Chronic liver disease	3533 (1.9)
9. Thyroid and endocrine diseases	3408 (1.8)
10. Hyperlipidemia	2791 (1.5)

^a^ICD-10: International Classification of Diseases 10th Revision.

### Statistical Analysis

Descriptive statistics were used to assess trends in the use of virtual visits and in-person visits. Continuous variables were described as the mean (SD). Categorical variables were described using frequencies and percentages.

Group comparisons (virtual visit vs in-person visit) were tested for differences using the Student t-test for continuous variables and the chi-square test for categorical variables. To determine the independent factors associated with the utilization of virtual clinics, all variables exhibiting a *P* value of <.01 on univariate analysis were entered into a multivariate binary logistic regression and a multilevel analysis. Multilevel analysis was conducted by using 3-level structure hierarchical linear modeling to incorporate variables at the patient/visit level, physician level, and department level in a statistically correct way.

All statistical analyses were carried out using commercially available software (IBM SPSS Statistics for Windows, version 28.0; IBM Corporation). The multilevel analysis was carried out using HLM version 8.2 (Scientific Software International). A two-sided *P* value of <.01 was considered statistically significant for all analyses.

## Results

### Visit Characteristics

Characteristics of all visits recorded during the study period are summarized in Table 2. Of 187,742 total visits, 10,927 (5.8%) were virtual visits during the study period. The mean age of patients in all visits was 61.48 (SD 16.86) years, and 96,884 (51.6%) visits were of female patients. In terms of the distance of the patients’ residence from the hospital, 168,846 (89.9%), 12,623 (6.7%), and 5284 (2.8%) visits were by patients who lived <20 km, 20-50 km, and >50 km from the hospital, respectively. In addition, 449 (0.3%) visits were by patients who needed to travel extensively to reach the hospital, including 167 (37.2%) visits by patients who lived in the outlying islands of Taiwan and 332 (62.8%) visits by patients who traveled >5 hours to reach the hospital. Nearly all (n=175,881, 93.7%) of the visits were reimbursed by the NHI. For disease type, 107,910 (57.5%) visits were related to a major chronic disease. Regarding specialty, 129,504 (69%) visits were conducted by the heart center, the psychiatry department, or the internal medicine department.

**Table 2:** Characteristics of all visits.

Characteristics		Total visits (N=187,742)		In-person visits (n=176,815)		Virtual visits (n=10,927)		Virtual visit rate (%)= virtual visits/total visits
Age (years), mean (SD); *P*=.013		61.48 (16.86)		61.50 (16.84)		61.09 (17.22)		N/A^a^
**Sex, n (%); *P*<.001**								
	Male	90,863 (48.4)	85,951(48.6)		4907 (44.9)		5.4	
	Female	96,884 (51.6)	90,864 (51.4)		6020 (55.1)		6.2	
**Distance, n (%); *P*<.001**								
	<20 km	168,846 (89.9)	159,787 (90.4)		9059 (82.9)		5.4	
	20-50 km	12,623 (6.7)	11,552 (6.5)		1071 (9.8)		8.5	
	>50 km	5284 (2.8)	4607 (2.6)		677 (6.2)		12.8	
	Outlying islands	167 (0.1)	133 (0.1)		34 (0.3)		20.4	
	Traveled >5 hours to reach the hospital	332 (0.2)	263 (0.1)		69 (0.6)		26.2	
	Unknown	490 (0.3)	473 (0.3)		17 (0.2)		3.5	
**Insurance type, n (%); *P*<.001**								
	Reimbursed by the NHI^b^	175,881 (93.7)	165,021 (93.3)		10,860 (99.4)		6.2	
	Nonreimbursed by the NHI	11,861 (6.3)	11,794 (6.7)		67 (0.6)		0.6	
**Disease type, n (%); *P*<.001**								
	Major chronic diseases	107,910 (57.5)	99,624 (56.3)		8286 (75.8)		7.7	
	Other diseases	79,832 (42.5)	77,191 (43.7)		2641 (24.2)		3.3	
**Department, n (%); *P*<.001**								
	Heart center, psychiatry department, and internal medicine department	129,504 (69.0)	120,088 (67.9)		9416 (86.2)		7.3	
	Other departments	58,238 (31.0)	56,727 (32.1)		1511 (13.8)		2.6	

^a^N/A: not applicable.

^b^NHI: National Health Insurance.

### Physician Characteristics

Of 174 invited physicians, 165 (94.8%) responded to the survey, accounting for 179,857 (95.8%) of 187,742 outpatient visits during the study period. These physicians were from 30 subspecialties of 13 departments of our hospital. The characteristics of the 165 physicians who responded to the virtual clinic service survey are summarized in Table 3. Their mean age was 55.58 (SD 11.89) years, 25 (15.2%) physicians were female, and 115 (69.7%) physicians expressed a positive attitude toward virtual clinics by agreeing or strongly agreeing (Likert scale score≥4) that virtual clinics are practical and that they are willing to conduct virtual visits.

**Table 3:** Physician characteristics.

Characteristics			Participants (N=165)
Sex (female), n (%)			25 (15.2)
Age (years), mean (SD)			55.58 (11.89)
Attitude (Likert scale score≥4), n (%)			115 (69.7)
**Department, n (%)**			
	Internal medicine	44 (26.7)	
	Surgery	37 (22.4)	
	Heart center	27 (16.4)	
	Obstetrics and gynecology	9 (5.5)	
	Oncology and radiotherapy	8 (4.9)	
	Psychiatry	8 (4.9)	
	Rehabilitation	6 (3.6)	
	Otorhinolaryngology	6 (3.6)	
	Ophthalmology	6 (3.6)	
	Dentistry	4 (2.4)	
	Dermatology	4 (2.4)	
	Pediatrics	4 (2.4)	
	Traditional Chinese medicine	2 (1.2)	

### Factors Associated With Virtual Clinic Utilization

In univariate analysis, the percentage of virtual clinic use was higher in female patients (6020/96,884, 6.2%, female patients vs 4907/90863, 5.4%, male patients, *P*<.001), in visits for major chronic diseases (8286/107,910, 7.7%, major chronic diseases vs 2641/79,832, 3.3%, nonmajor chronic diseases, *P*<.001), in visits reimbursed by the NHI (10,860/175,881, 6.2%, reimbursed visits vs 67/11,861, 0.6%, nonreimbursed visits, *P*<.001), and in visits performed by the heart center, the psychiatry department, or the internal medicine department (9416/129,504, 7.3%, visits in these departments vs 1511/58,238, 2.6%, visits not in these departments, *P*<.001). Patients who lived farther away from the hospital were more likely to use virtual clinic, with 69 (26.2%) of 332 visits by patients who needed more than 5 hours of travel time to reach the hospital, 34 (20.4%) of 167 visits by patients who lived in outlying islands of Taiwan, 677 (12.8%) of 5284 visits by patients who lived more than 50 km from the hospital, 1071 (8.5%) of 12,623 visits by patients who lived 20-50 km from the hospital, and 9059 (5.4%) of 168,846 visits by patients who lived within 20 km from the hospital (*P*<.001). There was no significant difference in the mean patient age between the in-person visit group (mean 61.50 years, SD 16.84 years) and the virtual visit group (mean 61.09 years, SD 17.22 years; *P*=.013).

Results of the 3-level structure multilevel analysis are shown in Table 4. The random part of the model represents the variance at each hierarchical level. Based on the formula of the intraclass correlation coefficient [21], physician-level factors accounted for 11.1% of the total variance and department-level factors accounted for 21.4% of the total variance in utilization of virtual clinics. Patient-/visit-level factors contributed to 67.5% of the total variance.

In multilevel analysis, patient-/visit-level factors associated with the utilization of virtual clinics included female sex (odds ratio [OR] 1.27, 95% CI 1.22-1.33, *P*<.001), distance from the hospital (OR 2.36, 95% CI 2.15-2.58 if distance>50 km, *P*<.001; OR 3.95, 95% CI 3.11-5.02 if extensive travel required, *P*<.001), visit for a major chronic disease (OR 1.33, 95% CI 1.24-1.42, *P*<.001), and visit reimbursed by the NHI (OR 7.29, 95% CI 5.71-9.30, *P*<.001). The physician’s age and sex were not associated with the utilization of virtual clinics. The only physician-level factor associated with the utilization of virtual clinics was the physician’s positive attitude toward virtual clinics (OR 1.50, 95% CI 1.16-1.93, *P*=.002). Department-level factors as represented by the different specialties were associated with the utilization of virtual clinics. The heart center, psychiatry department, and internal medicine department were more likely to use virtual clinics (OR 2.55, 95% CI 1.46-4.46, *P*=.004).

As shown in Table 4, combining all variables of different levels into a binary logistic regression model revealed that the physician’s age and sex were significantly associated with the utilization of virtual clinics. Therefore, in our study, the results may be skewed if binary logistic regression analysis were used, and the role of physician- and department-level factors in the utilization of virtual clinics (ie, factors beyond the patient/visit level) could not be assessed with such a model.

**Table 4:** Results of the multilevel model.

Variables			Logistic regression			3-level model			
			*β*	SE	*P* value	*β*	SE	OR^a^ (95% CI)	*P* value
**Fixed effect level 3**									
	Intercept		–5.322	0.144	<.001	–5.323	0.355	0.005 (0.002-0.011)	<.001
	Heart center, psychiatry department, and internal medicine department (yes/no)		0.630	0.035	<.001	0.936	0.254	2.550 (1.458-4.460)	.004
**Fixed effect level 2**									
	Physician’s sex, female (reference male)		0.119	0.031	<.001	0.193	0.180	1.214 (0.850-1.730)	.28
	Physician’s age		–0.004	0.001	<.001	–0.008	0.0050	0.992 (0.982-1.003)	.15
	Physician’s attitude, Likert scale score≥4 (yes/no)		0.234	0.024	<.001	0.404	0.129	1.498 (1.162-1.932)	.002
**Fixed effect level 1**									
	Patient’s sex, female (reference male)		0.228	0.020	<.001	0.242	0.020	1.274 (1.222-1.326)	<.001
	Insurance type, reimbursed by the NHI^b^ (yes/no)		2.030	0.130	<.001	1.986	0.124	7.288 (5.711-9.300)	<.001
	**Distance (reference <20 km and 20-50 km)**								
		>50 km	0.955	0.043	<.001	0.857	0.046	2.355 (2.153-2.577)	<.001
		Traveled extensively to reach the hospital	1.505	0.115	<.001	1.373	0.122	3.948 (3.108-5.016)	<.001
	Disease type, major chronic diseases (yes/no)		0.445	0.029	<.001	0.282	0.036	1.326 (1.236-1.421)	<.001
**Random effect**									
	Level 3		N/A^c^	N/A	N/A	1.040	1.020	N/A	<.001
	Level 2		N/A	N/A	N/A	0.541	0.736	N/A	<.001
	Level 1		N/A	N/A	N/A	*π*^2^/3	N/A	N/A	N/A

^a^OR: odds ratio.

^b^NHI: National Health Insurance.

^c^N/A: not applicable.

## Discussion

### Principal Findings

In our study, results of the multilevel analysis showed that factors at different levels all contributed to the utilization of virtual clinics. In addition to patient-/visit-level factors (67.5%), physician-level (11.1%) and department-level (21.4%) factors also drove variation in virtual clinic use. Patients who were female and lived farther away from the hospital were more likely to use virtual clinics, whereas the patient’s age did not affect the utilization of virtual clinics in our study. Visit-level variables, including insurance type (reimbursed by the NHI) and disease type (major chronic disease), were positively associated with the utilization of virtual clinics. The physician’s positive attitude toward virtual clinics positively predicted the use of virtual clinics, while the physician’s sex and age were not major predictors. Certain medical departments/specialties (the heart center, the psychiatry department, and the internal medicine department) were positively associated with the utilization of virtual clinics. Even when in-person visits and virtual visits were equally available, the use of virtual visits was relatively low, accounting for 10,927 (5.8%) of 187,742 visits in our study period.

### Comparison With Existing Literature

During the COVID-19 pandemic, a massive migration from in-person to virtual clinic visits was observed in many countries [2,4,22-24]. In prior reports from New York City, the epicenter of the pandemic during 2020, Mann et al [4] reported that telemedicine visits in a large academic health care system increased from less than 50 daily to more than 1000 daily, co-occurring with a decline of over 80% in in-person visits [4], and Ramaswamy et al [7] reported an 8729% increase in video visit utilization [7]. Various countries, including Italy, the United Kingdom, and India, reported virtual migration percentages between 60% and 95% of their usual practice [2,22]. However, during our study period, the COVID-19 outbreak in Taiwan was well controlled. No lockdown or shelter-in-place orders were enacted, and usual hospital outpatient services were not impacted. The use of virtual clinics was for individual health demand rather than for COVID-19–suspected diagnosis during our study period. Therefore, our hospital has not experienced a massive migration from in-person to virtual visits. Although there was a more than a 300-fold increase in the number of virtual visits compared to the prepandemic period, the majority (176,815/187,742, 94.2%) visits in our outpatient clinic were still in-person visits during the study period. This finding may imply that when both types of clinics are equally available, most patients still prefer in-person visits.

Earlier studies have reported that virtual clinics are associated with high patient/physician satisfaction [6-8,25,26], and findings have supported the effectiveness of virtual consultations in various practices [27-34]. Although teleconsultations or virtual clinics are not novel concepts, the growth of this type of health care service was slow in Taiwan owing to restrictive regulations prior to the COVID-19 pandemic. Our findings show that the utilization of virtual outpatient clinics was still limited even in the modern, high-income, capital city of Taiwan with a high penetration rate of smartphone use and broadband internet, implying that telemedicine may still have a long way to go to be widely accepted. Therefore, it is of great importance to investigate the factors impacting the utilization of virtual clinics in order to accelerate the implementation of telemedicine, especially in countries in which telemedicine services are currently underused.

Older age has been widely recognized as a barrier to adopting telemedicine in previous research [11,12,35-37]. However, no significant difference in patient age between the virtual visit and in-person visit groups was found in our study. Interestingly, some previous studies have found that age does not have a significant influence on a patient’s willingness to conduct telemedicine consultations [38,39], which may explain our finding. There are several additional possible reasons for our finding. First, as is common in Asian cultures, elderly patients are commonly cared for by family members who can provide assistance in using the telemedicine platform. Second, since chronic diseases were shown to be positively associated with the use of virtual clinics in our study, the higher prevalence of chronic diseases in the elderly may have contributed to their seeking of virtual clinic services. Finally, with the advances of smartphone technology, telemedicine is easier to use and more accessible than ever. Prior studies have shown that smartphone device usage is high even in people of older age [37,40]. For elderly people, lack of appropriate equipment and lack of exposure to new technology have been identified as significant barriers to adopting telemedicine [35]; therefore, the ease of use of the smartphone platform used in our study may be beneficial for elderly patients to complete a virtual visit.

Although patient sex was not associated with the utilization of telemedicine in some prior research [6,9,15], a few studies did show that being female is negatively associated with the success rate or satisfaction of a video visit. Eberly et al [13,14] reported that female patients are less likely to complete video visits, and Ramaswamy et al [7] found that female patients have lower satisfaction with video visits compared to male patients. Our study found that female patients are more likely than male patients to use virtual clinics. The same finding has been reported in some previous research that showed female sex as a positive predictor of digital health engagement behaviors and telemedicine consultations [11,36,41].

Prior studies have shown that time- and cost-saving benefits of telemedicine consultations are largely affected by distance from the hospital or clinic [30,42-44]. Cannon et al [45] found that for every 23 miles a patient resides from their clinic, patients are 111% more likely to use telemedicine consultation [45]. Our study showed that patients who live farther away from the hospital are more likely to replace in-person visits with virtual visits, which is consistent with previous research. We also found that patients with chronic and stable diseases are more likely to seek care by using virtual clinics. Since patients with chronic diseases and those who need to travel extensively to access health care are more likely to be vulnerable individuals, the implementation of virtual clinics is particularly meaningful and beneficial for these patient groups. Virtual health care may be even more essential during the COVID-19 pandemic in protecting these patients from disease exposure and decreasing the risk of infection.

Previous studies in the United States have shown that patients who use video visits are more likely to be White, have private health insurance, and have a higher level of income [13,15,36]. However, since the health care system is quite different in Taiwan, having private insurance was not a positive predictor of the utilization of virtual clinics in our study. In Taiwan, almost all citizens are covered by the NHI, and private insurance seldom covers the expense of outpatient visits. Interestingly, we found that patients are less likely to use virtual visits if the expense is paid out of pocket, which suggests that if the medical expense related to a visit is to be self-paid, patients would prefer an in-person visit with the physician.

Provider resistance has been reported by studies from Ethiopia, the United Kingdom, Australia, Iran, and the United States as a barrier to adopting telemedicine [35]. In our study, the physician’s age and sex did not influence the utilization of virtual clinics, but the physician’s positive attitude toward virtual clinics did (OR 1.50, *P*=.002), which supported the findings of those prior studies. We also found that in our institution, physicians of specific departments and subspecialties, including the Department of Internal Medicine, the Department of Psychiatry, and the heart center, are more likely to utilize virtual clinics than others. Since physician-level factors accounted for 11.1% and department-level factors accounted for 21.4% of the total variance in the utilization of virtual clinics, an increase in the physicians’ acceptance of virtual clinic services, particularly for physicians of certain departments, is important to further expand such service.

### Limitations and Strengths

This study had some limitations. First, demographic characteristics, including marital status, income, race/ethnicity, spoken language, and educational level, were not available in our database, so we were unable to capture the influence of these sociodemographic variables. However, although patients’ race/ethnicity and spoken language are common factors of patient inequities and disparities in telemedicine adoption, those factors may be not significant in Taiwanese society, as nearly all patients in Taiwan are ethnic Chinese. In addition, some prior studies examining various types of digital health utilization did not observe disparities in race/ethnicity [12,46].

Second, the practice and acceptability of telemedicine and virtual clinics may be strongly associated with the health care system structure and the pervasiveness of technology. The health care system of Taiwan is quite different from that in Western countries. A major difference is the lack of a well-established general practice and referral system in Taiwan. In large hospitals, physicians of various specialties also take on the role of primary care providers and have large volumes of outpatient visits for chronic diseases, such as diabetes, hypertension, hyperlipidemia, and coronary artery disease. The results of our study reflect this situation, with up to 107,910 (57.5%) of 187,742 visits during our study period having a principal diagnosis of a major chronic disease. However, our results may serve as a valuable reference for countries having a similar health care system structure, especially in East Asia.

Third, the study data were collected from a single hospital, limiting generalizability to different types of practices and organizations. However, to increase the relevance of study findings outside of the current institutional setting and practice, a multilevel analytic methodology was emphasized, which can be applied to different study cohorts and practice settings.

Despite these limitations, our study is unique in the following ways: First, unlike most of the virtual clinic studies conducted during the COVID-19 pandemic with lockdown or shelter-in-place orders and restrictions of regular medical services [6,7,12-15,36,47], our study was conducted with the background that virtual clinics and in-person clinics were both equally and easily accessible and the utilization of virtual clinics was not largely affected by the pandemic. Second, unlike most prior studies that focused only on patient-level variables that may affect the use of virtual clinics [12,13,36,37,48], we used multilevel analyses to show that insurance type, disease type, physician’s attitude, and specialties are associated with the utilization of virtual clinics independent of patient demographic characteristics. Third, many prior telemedicine studies were small and focused on specific patient populations of 1 specific medical specialty [6,9,10,13,25]. Our study was performed across various specialties and departments, and the patient volume of each virtual clinic was controlled by individual physicians; therefore, physician-level variables could be investigated. Finally, all our virtual visits were conducted using a user-friendly smartphone application platform; thus, we could greatly reduce the impact of technology-related barriers that may strongly impact the utilization of virtual clinics.

### Conclusion

In conclusion, our study demonstrates that factors at the patient/visit level, physician level, and department level all contribute to the utilization of virtual clinics. Female sex; residing at a greater distance away from the hospital; reimbursement by the NHI; seeking care for a major chronic disease; the physician’s positive attitude toward virtual clinics; and visits within certain departments, including the heart center, psychiatry, and internal medicine, were positively associated with the utilization of virtual clinics in our study. The findings may help direct future policy for expanding the use of virtual clinics, especially in countries struggling with the development and promotion of telemedicine virtual clinic services. Further studies should be conducted to evaluate the trends in and utilization of virtual clinics in the postpandemic period in different countries and health care systems, to examine the effectiveness and acceptability of telemedicine as a routine alternative practice to in-person clinics, and to investigate the economic impact of telemedicine from the perspective of the provider, the health care system, and society.

## Appendix

Multimedia Appendix 1 Questionnaire of the physician survey of virtual clinic service.

Multimedia Appendix 2 Classifications of chronic diseases defined by the Ministry of Health and Welfare of Taiwan.

Multimedia Appendix 3 Dictionary of specific ICD-10 codes used to categorize the major chronic diseases in this study.

## Abbreviations

EMR: electronic medical record
HIS: hospital information system
ICD-10: International Classification of Diseases 10th Revision
NHI: National Health Insurance
OR: odds ratio
