# Supplementary material for: Factors Associated With the Utilization of Outpatient Virtual Clinics: Retrospective Observational Study Using Multilevel Analysis
Source: J Med Internet Res. 2022 Aug 12;24(8):e40288. doi: 10.2196/40288 (PMC9377537; doi:10.2196/40288)
Supplement: Multimedia Appendix 1 [file jmir_v24i8e40288_app1.docx]

**Multimedia Appendix 1.** Physician survey of virtual clinic service

| We thank you in advance for completing this voluntary questionnaire. Your opinion is important for further improvement of our virtual clinic platform and service. | | | | | |
| --- | --- | --- | --- | --- | --- |
| Name: | | | | | |
| Employee Number: | | | | | |
| Please select the response that best describe your opinion/experience. | Strongly disagree | Disagree | Fair | Agree | Strongly agree |
| Part 1. Your general attitude about virtual clinic service: | | | | | |
| Q1-1. I feel that virtual clinic service is practical, and I am willing to conduct a virtual visit. |  |  |  |  |  |
| Part 2. Reliability of virtual clinic service: | | | | | |
| Q2-1. I have no doubt about the legality of a virtual visit. |  |  |  |  |  |
| Q2-2. I have no doubt about the data security of a virtual visit. |  |  |  |  |  |
| Q2-3. I have no doubt about the privacy of a virtual visit. |  |  |  |  |  |
| Part 3. Your experience about virtual clinics: | | | | | |
| Q3-1. I have ever conducted and completed a virtual visit.   - Yes (Please continue to answer part 4 – part 9 questions) - No (Please go to part 9) | | | | | |
| Part 4. Your confidence in your diagnoses and therapeutic plans in virtual visits: | | | | | |
| Q4-1. I was able to adequately communicate with patients. |  |  |  |  |  |
| Q4-2. Patients’ healthcare concerns were adequately addressed. |  |  |  |  |  |
| Q4-3. I was able to make proper diagnoses and therapeutic plans for my patients in virtual visits. |  |  |  |  |  |
| Part 5. Audio and video quality of our virtual clinic service: | | | | | |
| Q5-1. The video quality was adequate. |  |  |  |  |  |
| Q5-2. The audio quality was adequate. |  |  |  |  |  |
| Part 6. Design of our virtual clinic platform: | | | | | |
| Q6-1. The layout and visual design of the platform are appropriate. |  |  |  |  |  |
| Q6-2. The function of the platform is adequate. |  |  |  |  |  |
| Q6-3. The platform is easy to use. |  |  |  |  |  |
| Q6-4. Patients can pick up my video calls easily. |  |  |  |  |  |
| Q6-5. Overall, I am satisfied with the virtual clinic platform. |  |  |  |  |  |
| Part 7. Efficiency of virtual clinics: | | | | | |
| Q7-1. I feel that virtual visits are as efficient as in-person visits. |  |  |  |  |  |
| Q7-2. I completed all virtual visits successfully. |  |  |  |  |  |
| Part 8. Satisfaction of virtual clinic service: | | | | | |
| Q8-1. Overall, I am satisfied about the implementation of the virtual clinic service. |  |  |  |  |  |
| Q8-2. I would like to offer more virtual clinic service to my patients in the future. |  |  |  |  |  |
| Part 9. Comments and suggestions: | | | | | |
