# Supplementary material for: Factors Associated With the Utilization of Outpatient Virtual Clinics: Retrospective Observational Study Using Multilevel Analysis
Source: J Med Internet Res. 2022 Aug 12;24(8):e40288. doi: 10.2196/40288 (PMC9377537; doi:10.2196/40288)
Supplement: Multimedia Appendix 2 [file jmir_v24i8e40288_app2.docx]

**Multimedia Appendix 2.** The classifications of chronic diseases defined by the Ministry of Health and Welfare of Taiwan

| **Disease** | | Specific code for reimbursement | Corresponding major chronic disease group in Table 2 |
| --- | --- | --- | --- |
| **1. Cancer** | |  |  |
|  | Cancer | 12 | None |
| **2. Endocrine and metabolic disease** | |  |  |
|  | Thyroid dysfunction | 05 | Thyroid and endocrine disease |
|  | Diabetes mellitus | 01 | Diabetes mellitus |
|  | Hyperlipidemia | 19 | Hyperlipidemia |
|  | Wilson's disease | 48 | Thyroid and endocrine disease |
|  | Gout | 07 | None |
|  | Hyperprolactinemia | 43 | Thyroid and endocrine disease |
|  | Congenital metabolic disorders | 52 | Thyroid and endocrine disease |
|  | Endocrine disorders caused by adrenal gland | 70 | Thyroid and endocrine disease |
|  | Endocrine disorders caused by pituitary gland | 71 | Thyroid and endocrine disease |
|  | Precocious puberty | 72 | Thyroid and endocrine disease |
|  | Hypothyroidism | 80 | Thyroid and endocrine disease |
|  | Hypogonadism | 93 | Thyroid and endocrine disease |
| **3. Psychiatric disease** | |  |  |
|  | Psychiatric diseases | 47 | Psychiatric disease and sleep disorder |
| **4. Neurological disorders** | |  |  |
|  | Brain tumor complicated by neurological dysfunction | 73 | Cerebrovascular disease and other chronic neurologic diseases |
|  | Parkinson's disease | 16 | Cerebrovascular disease and other chronic neurologic diseases |
|  | Myotonic muscular dystrophy | 49 | Cerebrovascular disease and other chronic neurologic diseases |
|  | Other cerebral degeneration diseases | 54 | Cerebrovascular disease and other chronic neurologic diseases |
|  | Multiple sclerosis | 55 | Cerebrovascular disease and other chronic neurologic diseases |
|  | Infantile cerebral palsy and other paralytic syndromes | 56 | Cerebrovascular disease and other chronic neurologic diseases |
|  | Epilepsy | 15 | Cerebrovascular disease and other chronic neurologic diseases |
|  | Myasthenia gravis | 51 | Cerebrovascular disease and other chronic neurologic diseases |
|  | Polyneuropathy | 74 | None |
|  | Nerve root and plexus disorders | 75 | None |
|  | Trigeminal neuralgia | 76 | None |
|  | Migraines | 77 | Cerebrovascular disease and other chronic neurologic diseases |
|  | Spinal cord injury | 81 | None |
|  | Cerebrovascular disease | 14 | Cerebrovascular disease and other chronic neurologic diseases |
| **5. Cardiovascular disease** | |  |  |
|  | Cardiac diseases | 11 | Coronary artery disease, Chronic cardiac and arterial disease |
|  | Hypertension | 02 | Hypertension |
|  | Atherosclerosis | 57 | Chronic cardiac and arterial disease |
|  | Arterial thromboembolism | 58 | Chronic cardiac and arterial disease |
|  | Raynaud's disease | 26 | Chronic cardiac and arterial disease |
|  | Kawasaki disease | 78 | Chronic cardiac and arterial disease |
| **8. Chronic respiratory disease** | |  |  |
|  | Allergic rhinitis | 82 | Chronic respiratory disease |
|  | Chronic sinusitis | 45 | Chronic respiratory disease |
|  | Chronic bronchitis | 10 | Chronic respiratory disease |
|  | Emphysema | 20 | Chronic respiratory disease |
|  | Asthma | 06 | Chronic respiratory disease |
|  | Bronchiectasis | 22 | Chronic respiratory disease |
|  | Chronic obstructive pulmonary disease | 21 | Chronic respiratory disease |
|  | Pneumoconiosis | 59 | Chronic respiratory disease |
|  | Pulmonary disease caused by external causes | 60 | Chronic respiratory disease |
| **7. Disease of the digestive system** | |  |  |
|  | Peptic ulcer | 08 | None |
|  | Liver cirrhosis | 25 | Chronic liver disease |
|  | Chronic hepatitis | 03 | Chronic liver disease |
|  | Chronic cholangitis | 18 | None |
|  | Gastrointestinal functional disorders (including chronic pancreatitis, polyposis, irritable bowel syndrome, erosive gastritis, colitis) | 23 | None |
| **8. Diseases of the urinary system** | |  |  |
|  | Nephritis | 04 | None |
|  | Renal infection | 61 | None |
|  | Prostate hypertrophy | 66 | None |
|  | Chronic prostatitis | 94 | None |
|  | Urinary incontinence | 68 | None |
| **9. Diseases of the musculoskeletal system** | |  |  |
|  | Arthritis | 09 | None |
|  | Polymyositis | 50 | None |
|  | Osteoporosis | 27 | None |
|  | Lupus erythematosus | 24 | None |
|  | Chronic osteomyelitis | 95 | None |
|  | Dermatomyositis | 31 | None |
| **10. Ocular disease** | |  |  |
|  | Glaucoma | 33 | None |
|  | Keratoconjunctivitis sicca | 34 | None |
|  | Retinal degeneration | 35 | None |
|  | Macular degeneration | 36 | None |
|  | Uveitis | 37 | None |
|  | Vitreous hemorrhage | 38 | None |
|  | Corneal degeneration | 39 | None |
| **11. Infectious disease** | |  |  |
|  | Tuberculosis | 17 | None |
| **12. Congenital malformation** | |  |  |
|  | Congenital malformations | 62 | None |
| **13. Diseases of the skin and subcutaneous tissue** | |  |  |
|  | Psoriasis | 28 | None |
|  | Eczema | 32 | None |
|  | Leukoplakia | 83 | None |
|  | Seborrheic dermatitis | 84 | None |
|  | Amyloidosis | 85 | None |
|  | Pemphigoid | 86 | None |
|  | Herpetic dermatitis | 87 | None |
|  | Familial benign chronic pemphigus | 88 | None |
|  | Pemphigus | 30 | None |
|  | Onychomycosis | 29 | None |
|  | Hereditary epidermolysis bullosa | 89 | None |
|  | Ichthyosis | 90 | None |
|  | Keratosis pilaris | 91 | None |
|  | Progressive systemic scleroderma | 92 | None |
|  | Chronic urticaria | 98 | None |
|  | Atopic dermatitis | 99 | None |
| **14. Disease of blood** | |  |  |
|  | Chronic Anemia | 40 | None |
|  | Thrombocytopenia purpura | 41 | None |
|  | Hemophilia | 63 | None |
|  | Myelodysplastic syndrome | 96 | None |
|  | Essential thrombocythemia | 97 | None |
| **15. Diseases of ear** | |  |  |
|  | Chronic otitis media | 46 | None |
|  | Labyrinthitis | 44 | None |
|  | Tinnitus | AA | None |
| **16. Others** | |  |  |
|  | Follow-up after organ transplantation | 13 | None |
|  | Leprosy | 64 | None |
|  | Hemorrhoid | 65 | None |
|  | Endometriosis | 42 | None |
|  | Menopause syndrome | 67 | None |
|  | Polychlorinated biphenyls intoxication (69) | 69 | None |
|  | Congenital immunodeficiency | 53 | None |
|  | Human immunodeficiency virus infection | AB | None |
|  | Blackfoot disease | 79 | None |
